# Respiratory motion modelling for MR-guided lung cancer radiotherapy: model development and geometric accuracy evaluation

**DOI:** 10.1088/1361-6560/ad222f

**Published:** 2024-02-19

**Authors:** Björn Eiben, Jenny Bertholet, Elena H Tran, Andreas Wetscherek, Anna-Maria Shiarli, Simeon Nill, Uwe Oelfke, Jamie R McClelland

**Affiliations:** 1 Joint Department of Physics, The Institute of Cancer Research and The Royal Marsden NHS Foundation Trust, London, United Kingdom; 2 Centre for Medical Image Computing, Department of Medical Physics and Biomedical Engineering, University College London, United Kingdom; 3 Division of Medical Radiation Physics and Department of Radiation Oncology, Inselspital, Bern University Hospital and University of Bern, Bern, Switzerland; 4 Cambridge University Hospitals NHS Trust, Cambridge, United Kingdom; 5 Wellcome/EPSRC Centre for Interventional and Surgical Sciences, University College London, United Kingdom

**Keywords:** motion model, respiratory motion, motion management, IGRT, MR-Linac, MR-guided radiotherapy

## Abstract

*Objective.* Respiratory motion of lung tumours and adjacent structures is challenging for radiotherapy. Online MR-imaging cannot currently provide real-time volumetric information of the moving patient anatomy, therefore limiting precise dose delivery, delivered dose reconstruction, and downstream adaptation methods. *Approach.* We tailor a respiratory motion modelling framework towards an MR-Linac workflow to estimate the time-resolved 4D motion from real-time data. We develop a multi-slice acquisition scheme which acquires thick, overlapping 2D motion-slices in different locations and orientations, interleaved with 2D surrogate-slices from a fixed location. The framework fits a motion model directly to the input data without the need for sorting or binning to account for inter- and intra-cycle variation of the breathing motion. The framework alternates between model fitting and motion-compensated super-resolution image reconstruction to recover a high-quality motion-free image and a motion model. The fitted model can then estimate the 4D motion from 2D surrogate-slices. The framework is applied to four simulated anthropomorphic datasets and evaluated against known ground truth anatomy and motion. Clinical applicability is demonstrated by applying our framework to eight datasets acquired on an MR-Linac from four lung cancer patients. *Main results.* The framework accurately reconstructs high-quality motion-compensated 3D images with 2 mm^3^ isotropic voxels. For the simulated case with the largest target motion, the motion model achieved a mean deformation field error of 1.13 mm. For the patient cases residual error registrations estimate the model error to be 1.07 mm (1.64 mm), 0.91 mm (1.32 mm), and 0.88 mm (1.33 mm) in superior-inferior, anterior-posterior, and left-right directions respectively for the building (application) data. *Significance.* The motion modelling framework estimates the patient motion with high accuracy and accurately reconstructs the anatomy. The image acquisition scheme can be flexibly integrated into an MR-Linac workflow whilst maintaining the capability of online motion-management strategies based on cine imaging such as target tracking and/or gating.

## 1. Introduction

Respiration complicates the delivery of radiotherapy for lung cancer patients due to continuous target and organ at risk (OAR) motion. This motion is quasi-periodic meaning it shows inter- and intra-cycle variations as well as inter-fraction variation (McClelland *et al* 2013). Lung tumours and the surrounding anatomy can move by several centimetres (Seppenwoolde *et al* 2002). For optimal treatment, the general goal of radiotherapy is to deliver a highly conformal dose to the tumour whilst sparing healthy tissue and OARs with tight margins. To achieve the required dose distributions under motion conditions, active motion mitigation is needed which in turn relies on continuous motion monitoring (Bertholet *et al* 2019). MR-Linacs, with their ability of beam-on motion monitoring, promise to be an ideal tool to achieve these treatment goals given such demanding circumstances (Lagendijk *et al* 2008).

However, to leverage the full advantage of MR-guided radiotherapy (MRgRT), information of the volumetric, temporally varying patient anatomy needs to be generated which could then be used threefold. First, volumetric, time-resolved motion information could be used to increase delivery precision by guiding tracking and gating or a combination thereof. Second, it will enable accurate reconstruction of the delivered dose during beam-on. Third, the delivered dose reconstruction can then be used to inform down-stream treatment adaptation. Bertholet *et al* (2019) review real-time online motion monitoring and how it can be used for a range of motion mitigation as well as treatment plan adaptation strategies.

Any motion causes the anatomy to change over time. Hence, to obtain a volumetric representation of motion—e.g. in the form of an image or image series—four dimensions are required: three spatial dimensions and an additional one that represents the anatomical change (Stemkens *et al* 2018). For an accurate representation of arbitrary (i.e. non-periodic) motion, the last dimension should be time *t*. This is sometimes referred to as 3D + *t* but here we will use the term *time-resolved 4D*. Perfectly periodic motion—e.g. over-simplified and thus idealised breathing motion—on the other hand may be represented by selecting the fourth dimension as the phase of a breath cycle or its amplitude. These methods are referred to as *respiratory correlated 4D* images. Respiratory correlated imaging of breathing motion has the disadvantage, when compared to time resolved imaging, that it lacks the ability to capture inter- and intra-cycle variation in the observed motion.

It is currently not possible to acquire high-resolution time resolved 4D images with an MRI scanner, including MR-Linacs, that are sufficient for the aforementioned applications in MRgRT. As a result alternative approaches have been investigated in the literature. Most prominently, reducing the volumetric images to 2D slices centred on the target facilitates target motion monitoring in the imaging plane. 2D imaging is sufficiently fast for tracking and gating according to the recommendations of a maximum system latency of 500 ms (Paganelli *et al* 2018, Keall *et al* 2021). It can furthermore be interleaved with additional orthogonal or parallel image slices to estimate 3D target motion (Bjerre *et al* 2013, Sawant *et al* 2014, Paganelli *et al* 2015, Seregni *et al* 2016). Interleaved orthogonal slices were also used to perform motion-including dose reconstruction (Menten *et al* 2020). Therein the authors assumed clinical target volume (CTV) shifts and a rigid patient anatomy for prostate cancer radiotherapy treatments and thus making use of the information available during beam-on. However, 3D non-rigid out-of-plane motion away from the target remains inaccessible using these methods and accurate 3D delivered dose reconstruction based on the deforming anatomy is infeasible (Kontaxis *et al* 2017). Alternatively, respiratory correlated 4D images may be used, but all methods that rely on pre-sorting of data inherently disregard any inter-cycle variation in the observed motion pattern or require very long acquisition times (von Siebenthal *et al* 2007).

Respiratory, deformable motion models can address this issue by estimating the volumetric motion of the 3D anatomy on the basis of some dynamic (real-time) information. For this a static image of the anatomy is deformed using a pre-built model in conjunction with dynamic data (McClelland *et al* 2013). Beyond estimating the volumetric motion, these models are an efficient way to overcome the temporal constraints by fitting a model before application. Computationally expensive calculations are performed in a pre-processing task making the subsequent application substantially faster. Direct motion models describe the motion of interest as a function of one or more surrogate signals to compute a deformation. A surrogate signal is a one-dimensional, time-dependant signal that must be strongly related to the motion of interest and is often also referred to as a breathing trace. Examples of common surrogate signals are chest and diaphragm motion. Since surrogate signals ‘drive’ direct motion models during application, selecting appropriate signals must consider what type of data can be generated and accessed during the application phase (Tran *et al* 2020). Further consideration is required to determine the number of signals, since this determines the flexibility of the model. For breathing motion, at least two signals—or a signal and its temporal derivative—are required to describe intra-cycle variation, also known as hysteresis. Indirect motion models on the other hand optimise internal model parameters to fit the data.

Regardless of the flavour of motion models employed, a key requirement is the availability of initial data to fit the model. Commonly, data is pre-sorted (or binned) to overcome the temporal image acquisition constraints. Binning assembles information from different breath cycles into respiratory phase volumes to obtain a more or less coherent representation of the patient anatomy at each phase. Approaches proposed by Bjorn Stemkens *et al* (2016) or Huttinga *et al* (2022) and Huttinga *et al* (2023) sort k-space data, however, such sorting approximates the quasi-periodic nature of the breathing motion as perfectly repeatable motion. As a result, the base data used to build the motion models contains no information on how the motion can vary from breath-to-breath and are also prone to sorting artefacts.

In this study we build on the unified image registration and motion modelling framework by McClelland *et al* (2017) which does not rely on pre-sorting the input data and thus does not assume repeatable respiratory motion. The resulting models generate displacement vector fields (DVFs) from a surrogate signal to describe a motion state relative to a reference image and hence are fast in their application. This means that once the model is fully fitted it is able to provide time-resolved 4D information of the moving anatomy according to input surrogate signals in real time. The framework in general can handle a range of input data, e.g. image slices, thick slabs, projection data (Huang *et al* 2024), etc, but here we tailor it specifically to the use within an MR-Linac workflow. We present a flexible MR-acquisition scheme with two types of 10 mm thick 2D image slices as the building blocks, namely *motion slices* and *surrogate slices*. Motion slices have different locations and orientations to sample the full patient anatomy of interest and are used to model the 3D motion. Surrogate slices have a fixed location and orientation and are centred on the tumour. Surrogate and motion slices are interleaved. The surrogate signals are generated from the surrogate slices, and the model is fitted using the motion slices and surrogate signals as inputs. We furthermore alternate between model fitting and a super-resolution image reconstruction step and thus reconstruct a coherent, motion-compensated volume of the patient anatomy. One major benefit of the developed acquisition scheme is the similarity of the surrogate slices to the cine acquisitions currently used for target motion monitoring in clinical MR-Linac treatments. Hence, active motion mitigation such as gating and tracking can be performed parallel to the motion model data acquisition. However, the exact integration into a clinical workflow is beyond the scope of this paper.

As for all motion models, the geometric accuracy is difficult to quantify due to the lack of accurate knowledge on the ground truth motion and anatomy. Hence, first we use the anthropomorphic 4D extened cardiac-torso (XCAT) phantom and derive consistent and invertible DVFs (Segars *et al* 2010, Eiben *et al* 2020) to validate the models. Then, we demonstrate the feasibility of the tailored framework on eight clinical datasets acquired on an Elekta Unity MR-Linac (Elekta AB, Stockholm, Sweden) from four lung cancer patient volunteers.

## 2. Materials and methods

### 2.1. Motion modelling

**Figure 1.**
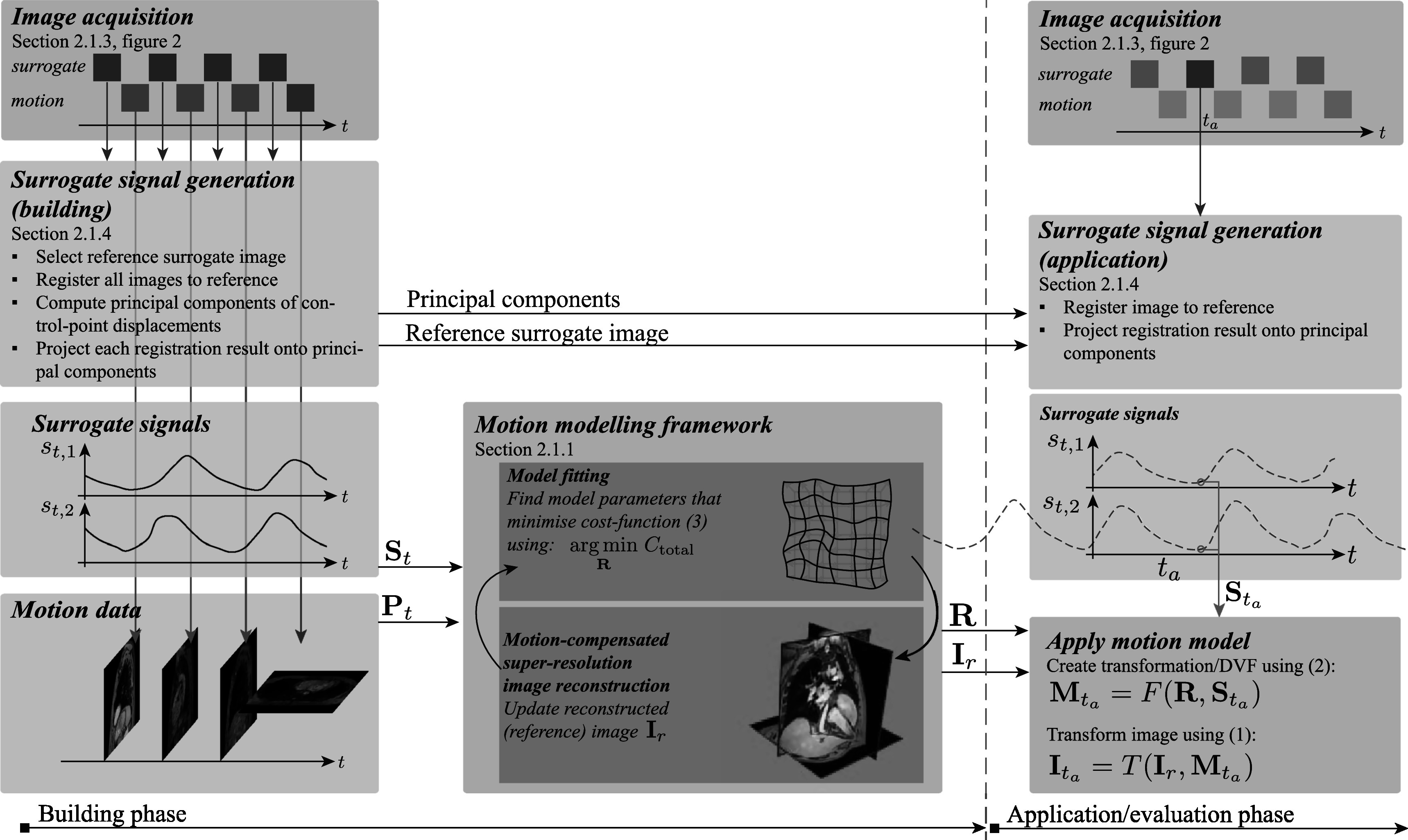
Flow chart illustrating how the motion modelling framework is used during the building and application phases. For the building phase surrogate and motion image slices are acquired in an interleaved way. A surrogate signal **S**_*t*_ is calculated from the surrogate slices and is used together with the motion data **P**_*t*_ (i.e. the motion slices) as an input to the motion modelling framework. The framework iterates between model fitting and motion-compensated super-resolution image reconstruction to output a fitted model **R** and an MCSRI **I**_*r*_. To apply the fitted motion model, a surrogate image is acquired at time point *t*_*a*_ from which a signal ${{\bf{S}}}_{{t}_{a}}$ is computed. This signal can then be used to calculate the transformation parameters ${{\bf{M}}}_{{t}_{a}}$ from the model **R**. This transformation is then used to deform the reconstructed image according to the motion estimated by the model for that time-point to ${{\bf{I}}}_{{t}_{a}}$. Details are provided in the referenced sections, equations, and figures.

This section introduces the motion modelling framework, its implementation, as well as the image data acquisition, and surrogate signal generation. The flow chart in figure 1 illustrates data that needs to be available before model fitting can commence, the components required to build the model during the building phase, and the data required to apply a fitted model in the application phase.

#### 2.1.1. Unified framework overview and open source implementation

The motion modelling framework used in this study was first presented by McClelland *et al* (2017) and unifies image registration and motion model fitting into a single optimisation procedure. This process can furthermore be alternated with an image reconstruction method to also reconstruct a motion-compensated reference image. Here, the overall motion modelling procedure is summarised first, followed by sections focussing on the specific implementations for MR-guided radiotherapy (see 2.1.3, 2.1.4).

In order to fit a model, the motion modelling framework takes dynamic image data as well as one or more respiratory surrogate signals as an input to reconstruct a single 3D reference image and a motion model. The dynamic image data has to be acquired fast enough to capture the motion and be free from motion artefacts such as blurring. However, the dynamic image data at each time point does not need to be a 3D image covering the full field of view (FOV), but can be ‘partial’ data such as one or a few slices with a limited FOV. The surrogate signals must have a strong relation with the internal motion being modelled (McClelland *et al* 2013). All data to build the model must be available before the model fitting and image reconstruction can commence. The fitted motion model generates an estimate of the subject’s internal motion as a function of the surrogate signals. During application, surrogate signals of a single time point are sufficient to create a motion estimate.

Assuming for now that the motion-free reference state image **I**_*r*_ is known, a function *T* deforms it for given motion parameters **M**_*t*_ to

\begin{eqnarray*}{{\bf{I}}}_{t}=T({{\bf{I}}}_{r},{{\bf{M}}}_{t}).\end{eqnarray*}

The subscript *t* indicates time dependence. This function encapsulates a spatial transformation and a suitable interpolation where the former is defined via a DVF ${{\bf{u}}}_{t\to r}^{{{\bf{M}}}_{t}}$, with the superscript indicating the DVFs’ dependence on the motion parameters, and the subscript indicating the direction of the vector elements pointing from a point **x** at time point *t* to the reference time point *r*. The transformation used here is the cubic B-spline based free-form deformation (FFD) where transformation parameters are defined on a regular control-point grid (Rueckert *et al* 1999). Hence, **M**_*t*_ are control-point displacements provided by the model for a time point *t*. The motion model **R** allows for calculation of the transformation parameters as a function *F* of the surrogate signals **S**_*t*_ with components *s*_*t*,*i*_. In this study the model is chosen to be a linear combination of its components **R**_*i*_

\begin{eqnarray*}{{\bf{M}}}_{t}=F({\bf{R}},{{\bf{S}}}_{t})=\displaystyle \sum _{i}{s}_{t,i}{{\bf{R}}}_{i},\end{eqnarray*}

where *i* denotes the surrogate signal component. To account for the image acquisition process, an operator *A*_*t*_ is defined. *A*_*t*_ simulates the acquisition of the measured (partial) image data, **P**_*t*_, from the full 3D image at time *t*, **I**_*t*_. For instance **P**_*t*_ could be x-ray projection data, in which case *A*_*t*_ would be the forward projection operator, or **P**_*t*_ could be a single thick slice, in which case *A*_*t*_ would be the slice selection profile. Since our acquisition scheme uses thick slices as dynamic image data (see section 2.1.3), we use a Gaussian slice selection profile with the full-width-half-maximum set to the acquired slice thickness. The adjoint of the image acquisition operator *A*_*t*_, ${A}_{t}^{* }$, for thick slices ‘spreads out’ the 2D slice image data over the corresponding voxels in the reference image space, according to the Gaussian slice profile. *A*_*t*_, and ${A}_{t}^{* }$ are time dependent to account for changes in the acquisition process such as slice orientation and position. The objective function *C*_total_ that is minimised during the model fitting to find the best model parameters is then constructed in line with other image registration procedures comprising a similarity ($\mathrm{Sim}(\cdot )$) and a constraint (or penalty) term (Con( · )). However, with the notable difference that the similarity term compares the acquired dynamic data **P**_*t*_ with the simulated dynamic data as estimated by the model (2) using the reference state image, the surrogate signal, the transformation, and the image acquisition operator, for all time points:

\begin{eqnarray*}{C}_{\mathrm{total}}=\displaystyle \sum _{t}\mathrm{Sim}({{\bf{P}}}_{t},{A}_{t}(T({{\bf{I}}}_{r},{{\bf{M}}}_{t})))+\lambda \mathrm{Con}({{\bf{M}}}_{t}).\end{eqnarray*}

The model parameters **R**_*i*_ are optimised by a conjugate gradient method and the transformation is a multi-resolution FFD (Rueckert *et al* 1999, Modat *et al* 2010) with optional bending energy (second-order) penalty as a regulariser and sum-of-squared differences as the similarity metric.

Up to this point the reference state image **I**_*r*_ was assumed to be known which however is not the case in general. Hence, we incorporate a motion-compensated image reconstruction into our algorithm by alternating between a reconstruction step and a model fitting step. The reconstruction algorithm calculates a volumetric reference state image **I**_*r*_ from all the (motion corrected) dynamic image data **P**_*t*_. To achieve this, the adjoint of the image acquisition operator *A* ^*^ is used to first transform the dynamic image data into the space of the reference image volume. The dynamic image data (in the space of the reference volume) is then deformed with the adjoint of the frunction *T*, *T* ^*^, to compensate for the motion estimated by the model for each acquisition time point *t*. To reconstruct a single, coherent image **I**_*r*_ from the deformed dynamic image data we use an iterative back-projection super resolution method (Irani and Peleg 1991). This algebraic super-resolution method requires thick, overlapping image slices to achieve a final isotropic resolution below the acquired slice thickness (see section 2.1.3). Since the model fitting step requires a reference state image to fit the model, and the reconstruction step requires a model **R** to motion-compensate the dynamic data, we start the alternation between fitting and reconstruction with model parameters that result in zero motion. Hence, the initial reconstruction generates an image with noticeable motion-induced artefacts, but still provides an initial approximation of the reference state image. This is followed by an update of the model parameters as described above resulting in an improved motion estimate, which in turn feeds into an updated reconstruction with reduced motion-induced artefacts. This procedure is repeated until convergence or a specified number of iterations is reached.

The result of the model building phase is a motion-compensated, reconstructed reference state image **I**_*r*_ and the fitted model parameters **R** (2). In a subsequent application (or evaluation) phase **R** can be used to estimate the motion of the patients’ anatomy relative to **I**_*r*_ at any time point for which surrogate signals **S** can be provided. In a real-time application scenario, the fitted model could be used to prospectively estimate the patients’ motion.

#### 2.1.2. Open-source implementation

The motion modelling framework was implemented in an open source package called *SuPReMo*, for Surrogate Parametrised Respiratory Motion Modelling and is available online (SuPReMo 2021). The class structure reflects the components outlined in section 2.1.1, such as *optimiser*, *transformation*, *correspondence model*, *image acquisition* etc and was designed with the goal of extensibility for research purposes. Continuous integration testing enables validation against a reference research implementation and a detailed documentation is provided alongside the repository. All motion models were fitted and motion-compensated super resolution images (MCSRIs) reconstructed with the parameters detailed in the appendix A.1.

#### 2.1.3. Interleaved acquisition geometry

**Figure 2.**
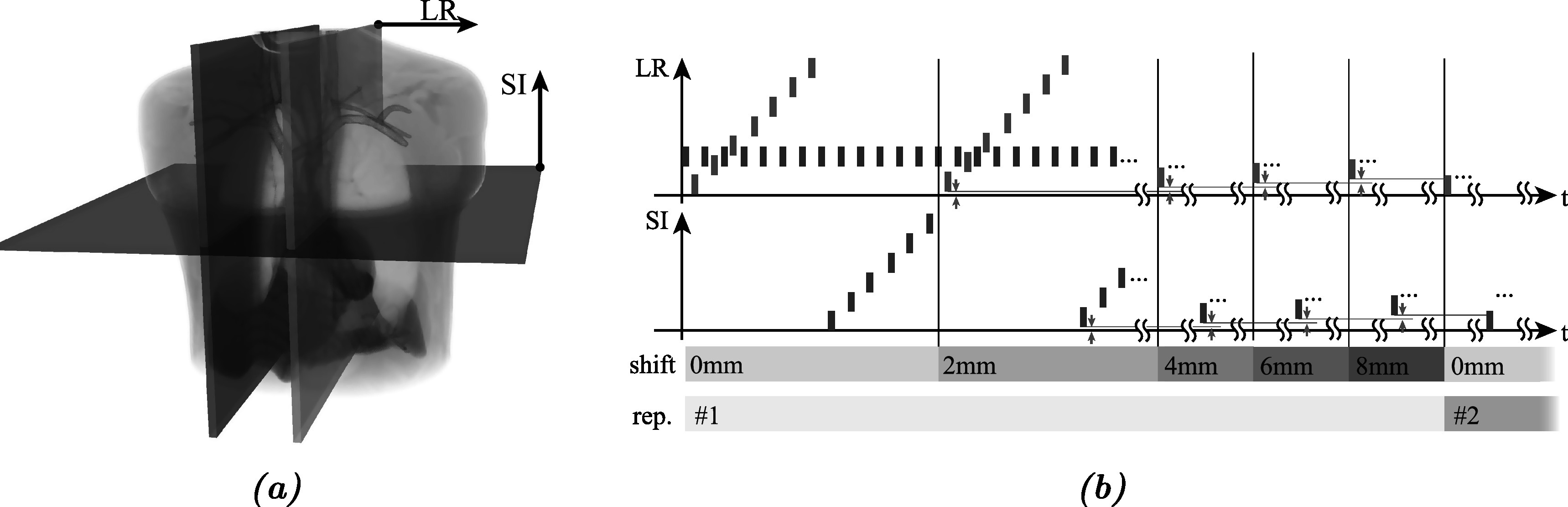
Orientation (a) and temporal order and position (b) of slices used in the acquisition scheme. Surrogate slices (blue) are interleaved with motion slices which first cover the anatomy from left to right (green), then from inferior to superior (red). Thereafter, the motion slice origin is offset by 2, 4, 6, and 8 mm (to enable iterative back-projection super-resolution image reconstruction) and the acquisition is continued after each shift. A set of five shifts completes a repetition.

In this study we use a sequence of 10 mm thick MR slices in different orientations and locations. To fit the motion model, the values of the surrogate signals need to be available for each dynamic image. Therefore, we interleave the acquisition of surrogate slices, which are used to calculate the surrogate signal values (see section 2.1.4), and motion slices, which are used as the dynamic images. The surrogate signals for the motion slices can then be interpolated from the surrogate slices. Figure 2 illustrates the orientation and temporal slice order.

*Surrogate slices* have a sagittal orientation and are positioned centrally on the tumour, i.e in a fixed location (shown in blue in figure 2). This enables generation of a consistent, low-dimensional surrogate signal. While surrogate slices are used to generate the surrogate signal **S**_*t*_, *motion slices* provide information about the anatomy and its motion. These slices are input to the framework as dynamic image data **P**_*t*_ (see section 2.1.1) and are acquired as follows. First, sagittal slices (green) sample the FOV form left to right, followed by axial slices (red) form inferior to superior. Once the complete FOV has been sampled by motion slices of both orientations, the origin of these slices is shifted by 2 mm orthogonal to the imaged plane and the acquisition is continued (see figure 2(b)). After five shifts (0, 2, 4, 6, and 8 mm) the original imaging geometry is reached again, completing a *repetition*. The partially overlapping thick slice acquisition enables the use of an iterative back-projection super resolution method for the motion-compensated image reconstruction resulting in an MCSRI (Irani and Peleg 1991, McClelland *et al* 2017). Multiple repetitions can be acquired and used in the building phase to improve the quality of the MCSRI and the accuracy of the motion model.

#### 2.1.4. Surrogate-signal generation

Tran *et al* (2020) performed an extensive evaluation of various image-based respiratory surrogate signals derived from 2D surrogate slices as used in this work. The surrogate signals that resulted in the most accurate of the compared models were generated by first performing 2D deformable image registration on the surrogate slices, and then applying principal component analysis (PCA) on the registration results and using the first two principal components as surrogate signals. Therefore, this method was used to derive the surrogate signals in this work. A summary of the different steps involved is outlined below.

To generate consistent surrogate signals for the complete duration of the experiment including model building and subsequent application (or evaluation), all surrogate slices from building and evaluation must be registered to a common reference image. This image is selected from the surrogate slices of the first shift of the first repetition and a slice close to the mid-position of the breathing cycle is automatically selected based on the image intensities. The registrations were performed using the open-source software Nifty-Reg (Modat *et al* 2010, 2020) with the parameters given by Tran *et al* (2020). PCA was performed on the registration results, i.e. on the displacements of the control points that define the FFDs used in NiftyReg, and the first two principal components were used as the surrogate signals. The surrogate signals were normalised to the vector median value (Astola *et al* 1990) of the building phase and the time-point where the median was reached was defined as *t*_*r*_, i.e. after normalisation we get ${{\bf{S}}}_{{t}_{r}}={\bf{0}}$. This means the reference state image will correspond to the time-average (median) position of the anatomy over the acquisition. Since the vector median is an element of the original set we guarantee that the reference position was reached during the building phase.

### 2.2. Simulated and patient data evaluation

To thoroughly evaluate the geometric accuracy of our motion models we used both simulated data and real data from volunteer patients. For both the simulated and real data, a total of five repetitions (see section 2.1.3) were used, corresponding to ∼20 min acquisition (∼4 min per repetition, range 220–240 s). The first three repetitions were used for building the models, and the next two for evaluation.

#### 2.2.1. Simulated ground-truth data

Simulated data provides a valuable tool for evaluating the motion models, since the ground truth motion is known, which is not possible for real patient data. The simulated ground truth motion can be compared to the motion estimated by the model and used to quantitatively assess the geometric accuracy of the models. However, generating this data is not trivial since it should resemble the application scenario as close as possible whilst providing a convincing degree of realism. To achieve this, we used the XCAT phantom (Segars *et al* 2010) in combination with the publicly available post-processing framework *cid-X* (Eiben *et al* 2020, cid-X 2021) to generate consistent and invertible DVFs as ground truth motion. Notably, these DVFs include sliding motion comparable to real organ motion. In the processing framework only a single anatomical image **I**_GT_ is generated for the first time point of the simulation. For all subsequent time points, consistent and invertible forward and backward DVFs from the first to the current time point ${{\bf{u}}}_{0\to t}^{\mathrm{GT}}$ and their inverse ${{\bf{u}}}_{t\to 0}^{\mathrm{GT}}$ are generated, and the anatomy is deformed accordingly. Hence, by the means of composition, the motion between any simulated time points *t*_*a*_, and *t*_*b*_ can be calculated using

\begin{eqnarray*}{{\bf{u}}}_{{t}_{a}\to {t}_{b}}^{\mathrm{GT}}({\bf{x}})={{\bf{u}}}_{0\to {t}_{b}}^{\mathrm{GT}}({{\bf{u}}}_{{t}_{a}\to 0}^{\mathrm{GT}}({\bf{x}})).\end{eqnarray*}

**Figure 3.**
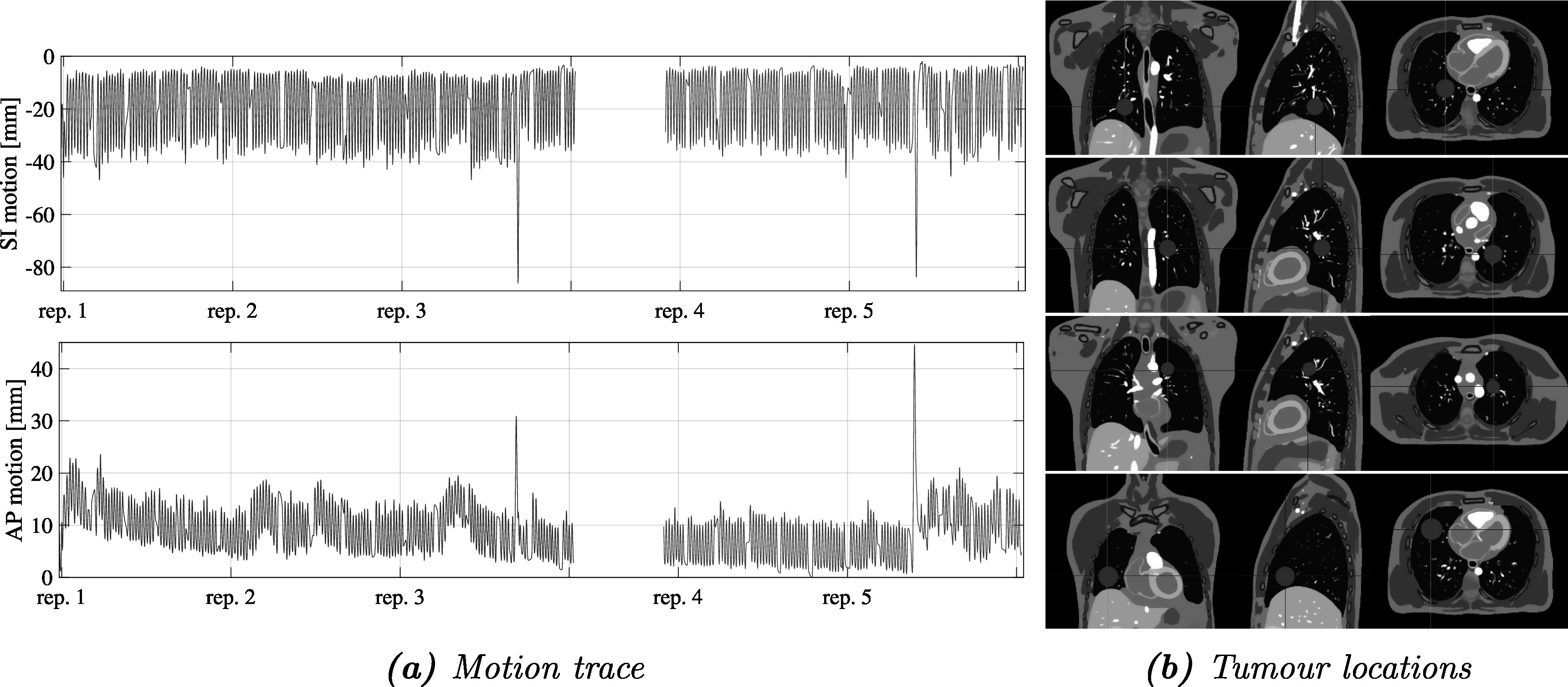
Motion trace acquired from a volunteer cine-MRI scan used to animate the XCAT phantom motion (a). Blue and orange traces indicate the parts used for building (repetitions 1–3) and evaluating (repetitions 4–5) the motion modelling framework, respectively. The simulated lung tumour locations (A, B, C, and D) within the XCAT anatomy highlighted in red (b).

The motion traces driving the simulations were extracted from a 30 min cine-MR acquisition of a healthy volunteer within which the chest and diaphragm motion was tracked (figure 3(a)). These two measurements that include realistic inter- and intra-cycle variations were input into the XCAT phantom to generate the DVFs, which were then post-processed as outlined above. Four anatomical images with MR-like contrast and varying tumour locations were generated resulting in four time series with known volumetric deformations (figure 3(b)).

The volumetric time series were used to simulate the interleaved thick-slice data acquisition as described in section 2.1.3. A Gaussian slice-selection profile was applied to the volumetric data to extract the required slices and finally Rician noise was added to achieve a simulation visually comparable to the data acquired with the MR-linac. Surrogate slice locations were selected according to the four different simulated tumour positions and surrogate signals were calculated as described in section 2.1.4.

#### 2.2.2. Motion compensation evaluation

Reconstructing a high-quality MCSRI requires a good estimate of the motion, which is equivalent to a well-fitted motion model. To evaluate the effectiveness of the motion compensation provided by the model, we introduce the *zero model* where the motion is set to zero for all time points and locations, i.e. ${{\bf{u}}}^{{{\bf{M}}}_{t}}({\bf{x}}):= {\bf{0}}$—which is equivalent to an unfitted model that always estimates no motion. Then, the image reconstruction is performed with the zero model first using intensity averaging to reconstruct an average image (AVGI) and second using super-resolution image reconstruction via iterative back-projection to reconstruct a super-resolution image (SRI). For comparison, the full model fitting and MCSRI reconstruction as described in section 2.1.1 is performed, and all reconstructed images are compared against the known ground truth for the simulated data visually and in terms of the mean absolute error (MAE) of the image intensities.

#### 2.2.3. Geometric accuracy with known ground truth

The deformation field error (DFE) is calculated for a position **x** and a time point *t* as the l2-norm of the difference between the known ground truth deformation **u** ^GT^ and the model estimated one ${{\bf{u}}}^{{{\bf{M}}}_{t}}$ using

\begin{eqnarray*}{\mathrm{DFE}}_{t}({\bf{x}})={\parallel \left({{\bf{u}}}_{t\to {t}_{R}}^{{{\bf{M}}}_{t}}-{{\bf{u}}}_{t\to {t}_{R}}^{\mathrm{GT}}\right)({\bf{x}})\parallel }_{2}.\end{eqnarray*}

The spatial mean over all voxels within the patient DFEs were calculated for all time points from the evaluation repetitions, and summarised using the temporal mean, standard deviation, and 95th percentile over all time points. In addition, summary statistics were calculated for other regions of interests (ROIs), i.e. the tumour, lungs, heart, and liver, to evaluate the performance of the model in specific ROIs. 3D maps of the mean DFE over all evaluation time points were generated to visualise the spatial distribution of the DFE. For comparison, the DFE was also calculated with the zero model.

#### 2.2.4. Patient data

The proposed motion models have also been evaluated on data from volunteer patients to demonstrate that they can be successfully applied to real data. Patients were consented under the PRIMER study, approved under IRAS: 208 449 and REC: 17/LO/0907. Four patients who were treated on standard linacs at our institution were scanned on a 1.5 T Elekta Unity MR-Linac (Elekta AB, Stockholm, Sweden). The first two patients were scanned on three different treatment days, hence, a total of eight patient datasets were available. Patient details are given in table 1. For patient comfort the scans were acquired with arms down.

**Table 1.** Clinical details of patients included in this study. Non-small cell lung cancer (NSCLC) and number of datasets acquired per patient (*N*).

case	age	sex	TNM-stage	diagnosis and tumour location	*N*
p1	47	male	T4 N3 M0	locally advanced adenocarcinoma of the left upper lobe with bilateral hilar and mediastinal nodes, NSCLC	3
p2	71	male	T1b N3 M0	locally advanced adenocarcinoma of the left upper lobe with mediastinal and contralateral supraclavicular node, NSCLC	3
p3	79	male	T4 N0 M0	locally advanced squamous cell carcinoma of the right lower lobe, tumour associated with distal collapse (atelectasis)	1
p4	85	female	T1c N0 M0	Early stage adenocarcinoma of the left upper lobe, (early stage), NSCLC	1

The 10 mm thick motion and surrogate slices were acquired using the pattern described in section 2.1.3 using a gradient echo T1-weighted sequence. An echo and repetition time of TE = 2.08 ms and TR = 4.29 ms respectively and a flip angle of 10° were selected. The in-plane resolution of the reconstructed slices was 2 × 2 mm^2^ and the image matrix was 160 × 160 voxels. Each repetition comprised 330 surrogate and motion slices respectively and consecutive images were acquired with a frame rate between 2.7 and 3 Hz. The acquisition of a single repetition for patient p1 took 240 s; it was 220 s for all other patients.

#### 2.2.5. Geometric accuracy without known ground truth

For patient data, the ground-truth motion is unknown making direct evaluation of the DFE impossible. Hence, we estimate the geometric accuracy of the model by performing a *residual error registration* between the acquired motion slices and the model estimated equivalents—i.e. the MCSRI is deformed by the model and the corresponding 10 mm thick slice is simulated using a Gaussian slice-selection profile. The registrations measure the 2D displacements in the imaging plane required to align the model estimated slices with acquired motion slices, which we call the *residual error*. They cannot account for displacements in the through slice direction, and as such do not directly estimate the 3D geometric accuracy of the model as the DFE does. But in the absence of ground truth motion the residual error provides a useful way to estimate the geometric accuracy. To investigate how well the residual error approximates the DFE, the residual error registrations were also performed for the simulated data.

We used NiftyReg ((Modat *et al* 2020) to register the images and generate corresponding displacement vector fields (registration parameters are given in detail in the appendix A.2). The results were summarised as described in section 2.2.1 for the DFE. The summary statistics were calculated separately for the model building and evaluation repetitions.

## 3. Results

Motion models were built from the simulated and patient data. The fitting and reconstruction parameters are given in appendix A.1. Furthermore, animations of the model results (first simulated dataset and one per patient volunteer) are provided as supplementary materials and described in appendix A.3.

The duration to fit the models and reconstruct the MCSRIs for the patient data was on average 40.3 min (range 38.2–43.4 min). To generate the surrogate signals, the computationally most expensive operation is the 2D registration of the current surrogate image to the reference surrogate image. We measured the time of each 2D registration for the first patient to take on average 379 ± 62 ms. The model application time (see box named *Apply motion model* in figure 1) was measured for 100 time points. For each time point a DVF and a volumetric deformed image was generated. This took on average 42.1 ± 1.3 ms per application time point. All time measurements were preformed on an Intel Core i9-10850K with ten cores and 64 GB of RAM

### 3.1. Simulated data

**Figure 4.**
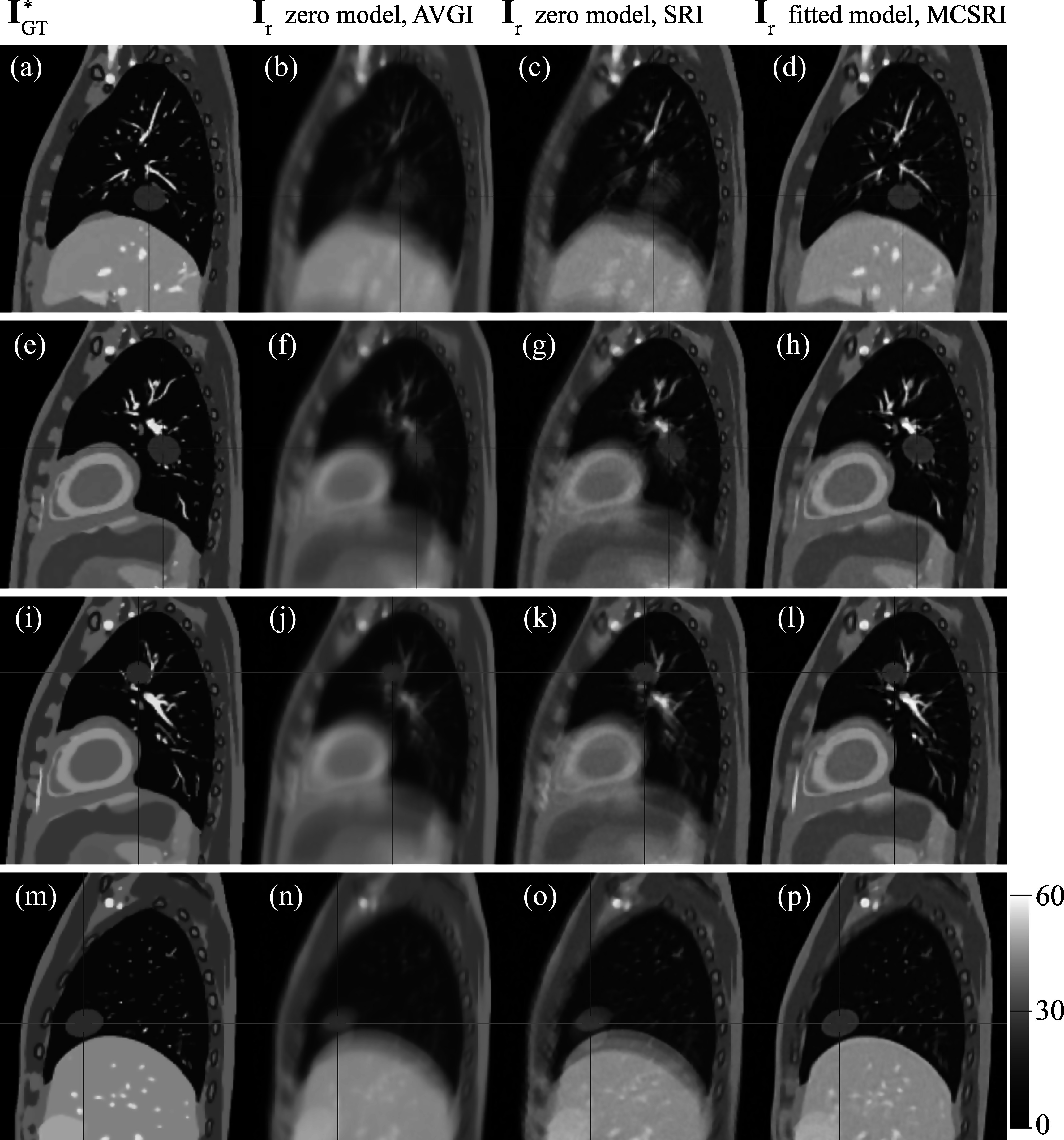
Comparison of the ground-truth images with non-motion compensated image reconstructions AVGI, and SRI, and the MCSRIs output by our proposed algorithm, using the motion model for motion-compensation. Each row represents the results of a simulated tumour position (A–D).

Figure 4 shows sagittal slices of the ground truth images transformed to the reference time point (${{\bf{I}}}_{\mathrm{GT}}^{* }$first column, (a, e, i, m)), the reconstructed but not motion-compensated images using the zero model and either intensity averaging, AVGI (second column, (b, f, j, n)), or super resolution reconstruction, SRI (third-column, (c, g, k, o)). The MCSRIs generated with our proposed combined model fitting and motion-compensated super-resolution reconstruction algorithm are shown in the fourth column, (d, h, l, p). Severe blurring can be observed in the AVGIs due to the thick, overlapping slices and averaging over all acquisition time points whilst the anatomical motion is ignored. The super-resolution reconstruction without motion compensation generates substantially clearer image details in anatomical regions that do not move noticeably in the simulation, visible in superior, posterior regions. However, blurring and duplication of structures—such as the tumour, diaphragm, and vessels inside the lung—can still be observed in moving parts of the anatomy. This is particularly evident further inferior due to the larger motion amplitude in these regions. The motion compensation provided by the fully fitted model in combination with the super-resolution image reconstruction results in an excellent recovery of image details and MCSRIs are in close agreement with the simulated ground truth images. The mean (range) MAEs between the ground truth and the reconstructed images are 2.23 ([2.21; 2.23]), 1.86 ([1.85; 1.87]), 1.41 ([1.40; 1.43]) for the AVGIs, the SRIs, and the MCSRIs respectively.

**Figure 5.**
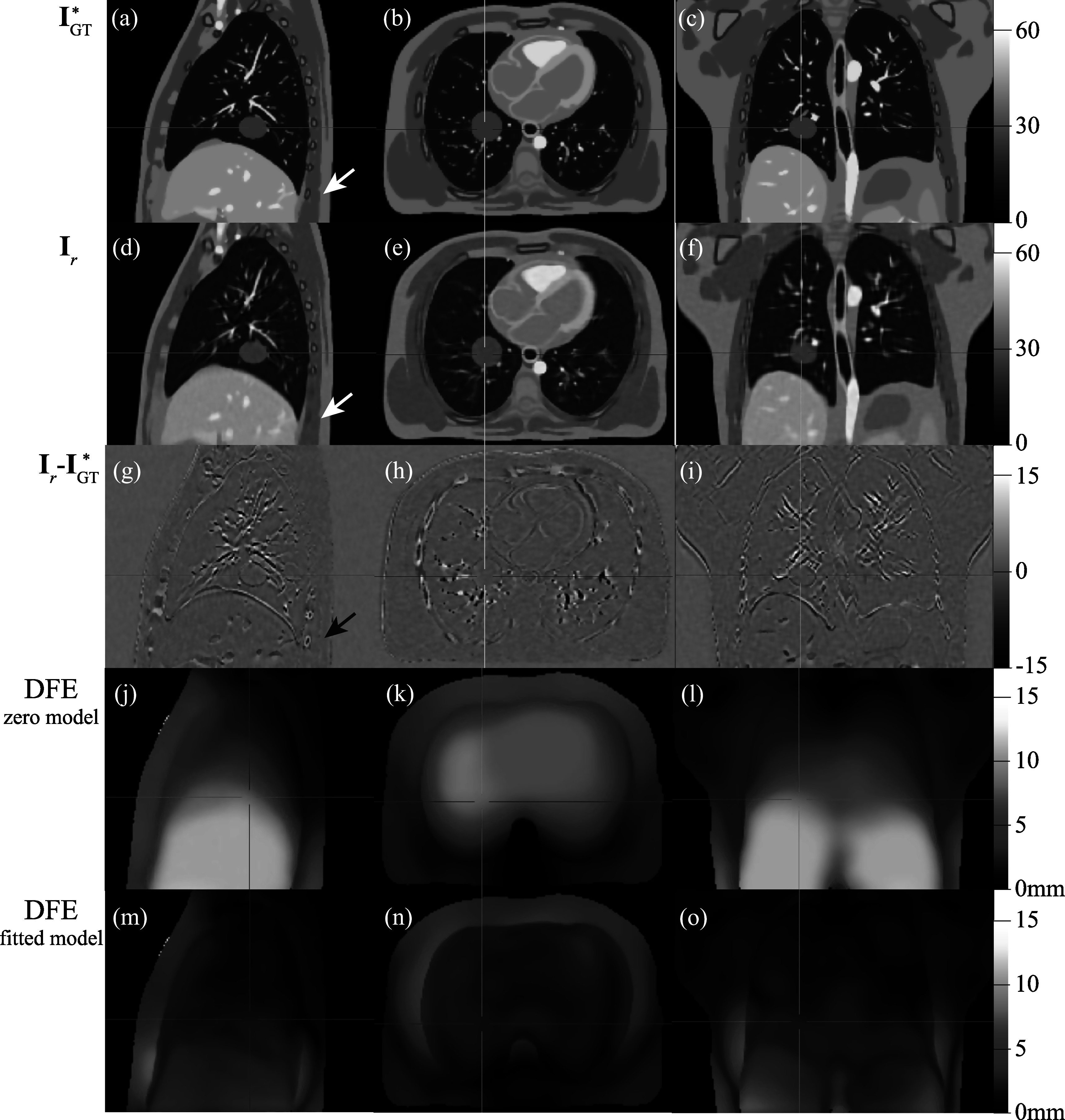
Sagittal, axial, and coronal slices of the ground truth image volume transformed to the selected reference time point (the asterisk indicates that the deformed ground-truth image ${{\bf{I}}}_{\mathrm{GT}}^{* }={{\bf{I}}}_{\mathrm{GT}}({{\bf{u}}}_{{t}_{R}\to 0}^{\mathrm{GT}}({\bf{x}}))$ is shown, (a), (b), (c)), volumetric MCSRI volume **I**_*r*_ ((d), (e), (f)), the difference between these images ((g), (h), (i)). The bottom two rows visualise the magnitude of the mean DFE over all evaluation time points measured against the simulated ground truth motion when the zero model is assumed ((j), (k) (l)) and when the motion is estimated by the fully fitted model ((m), (n) (o)).

Figure 5 compares the MCSRI produced by our proposed method for simulation A to the corresponding ground truth image. The first row shows a sagittal, axial and coronal slice of the ground truth image (a, b, c), the second row shows the same slices of the MCSRI **I**_*r*_ (d, e, f), and the third row shows the difference image between the previous two rows (g, h, i). The MCSRI closely resembles the ground truth anatomy and preserves the overall contrast. Some slight blurring can be observed in places such as the diaphragm and vessels within the lungs. A notable loss of detail of the lower posterior ribs can be seen in the lower right of figure 5(a), (d), (g) as indicated by the arrows. This reconstruction artefact occurs where the simulated ground truth anatomy exhibits substantial sliding motion.

The bottom two rows of figure 5 show the spatial distribution of the mean DFE over all evaluation time points. Figures 5(j), (k), (l) show the results for the zero model, i.e. ${{\bf{u}}}^{{{\bf{M}}}_{t}}={\bf{0}}$. Figures 5(m), (n), (o) show the results when the fully fitted model is used to estimate the motion. A substantial drop in mean DFE can be observed throughout the anatomy with a localised error remaining in the sliding regions, which agrees with the blurring seen in the MCSRI in the same regions. Table 2 summarises the mean DFE in terms of mean, standard deviation and 95th-percentile over all evaluation time points for all simulated cases within various ROIs, namely the patient contour (body), the tumour, the lungs, the liver, and the heart. The results in the section ‘zero model’ directly measure the simulated motion and thus provide a statistical summary of the simulated motion.

**Table 2.** Mean deformation field error (DFE) measured for various regions of interest (i.e. body, tumour, lungs, liver, and heart, in mm) against the ground truth motion during the testing phase for the four simulated tumour positions (A, B, C, and D) when the model estimates no motion (zero model, top rows) and when the model is applied (model, bottom rows). Note: A different reference image was automatically selected for tumour position B compared to positions A, C, and D (see section 2.1.4) leading to slightly different values for the common structures of the zero model.

Mean DFE [mm]															
	mean					std.					95th—%*ile*				
	tumour	body	lungs	liver	heart	tumour	body	lungs	liver	heart	tumour	body	lungs	liver	heart
*zero model*				
A	8.11	3.84	3.82	10.56	7.33	0.83	3.65	2.77	0.75	0.54	9.47	10.88	9.21	10.91	8.01
B	4.11	3.90	3.89	10.75	7.46	0.84	3.70	2.82	0.71	0.55	5.56	11.07	9.37	11.10	8.15
C	2.27	same as A				0.32	same as A				2.83	same as A			
D	7.20	same as A				1.35	same as A				9.42	same as A			

*model*				
A	1.13	1.63	0.91	3.41	1.17	0.17	1.56	0.55	1.79	0.39	1.38	4.90	1.81	7.25	1.96
B	0.74	1.72	1.08	3.53	1.28	0.14	1.62	0.66	1.85	0.41	0.98	5.09	2.24	7.44	2.10
C	0.31	1.59	0.88	3.29	1.08	0.05	1.55	0.54	1.75	0.40	0.41	4.85	1.76	7.04	1.90
D	0.95	1.59	0.86	3.35	1.11	0.19	1.55	0.52	1.71	0.41	1.27	4.85	1.73	7.02	1.92

It can be seen that the DFEs for the model are all substantially reduced compared to the zero model. The mean DFEs over the whole body are below 2 mm (the voxel size) and the 95th percentile is below 5 mm except for tumour position B, where it is 5.09 mm. The DFEs for the tumour, lungs, and heart, which are the most important structures for lung RT, are all even lower, with 95th percentiles of 2.24 mm or less. The larger DFEs for the liver are due to the sliding motion between the liver and the surrounding ribs. Moreover, parts of the liver are very homogeneous with little structure to guide the model fitting in these areas. Higher errors are mostly observed at the edge of the FOV.

Table 3 shows the DFEs measured within the 3D patient anatomy and split into different anatomical directions (superior-to-inferior (SI), anterior-to-posterior (AP), and left-to-right (LR)) for better comparison with the results produced by the residual error registration. All models perform similarly with the highest mean error in the direction of the largest initial motion amplitude (SI).

**Table 3.** Mean deformation field error measured for all testing time points within the modelled anatomy. Model estimates are measured against known ground-truth deformations and results are given for different anatomical directions in mm.

Mean DFE, model versus ground-truth [mm]									
	mean			std.			95th—%*ile*		
	SI	AP	LR	SI	AP	LR	SI	AP	LR
A	1.33	0.61	0.31	1.05	0.43	0.15	2.31	1.09	0.54
B	1.39	0.67	0.30	0.78	0.44	0.16	2.20	1.17	0.51
C	1.29	0.60	0.30	0.80	0.41	0.16	2.27	1.07	0.50
D	1.31	0.59	0.30	0.81	0.42	0.17	2.28	1.06	0.49

Table 4 gives the results of the residual error registrations for the simulated data. The largest residual errors are observed in the SI direction followed by AP and then LR directions. Calculating the l2-norm of the mean residual error vectors for each simulated case results in 1.65 mm, 1.67 mm, 1.64 mm and 1.64 mm respectively. This closely resembles the mean DFE measured within the body contour in table 2 which ranges between 1.59 and 1.72 mm. A comparison with table 3 shows that the residual error registration slightly underestimates the mean DFE in the SI direction as well as its variability and 95^th^ percentile.

**Table 4.** Result of the residual error registration for the four simulated tumour position (evaluation phase), values given in mm.

Mean residual error, model versus simulated acquisition [mm]									
	mean			std.			95th—%*ile*		
	SI	AP	LR	SI	AP	LR	SI	AP	LR
A	1.25	0.84	0.68	0.33	0.29	0.20	1.93	1.32	1.06
B	1.27	0.84	0.68	0.35	0.29	0.20	1.99	1.32	1.06
C	1.24	0.83	0.67	0.33	0.29	0.19	1.94	1.31	1.04
D	1.25	0.83	0.67	0.33	0.31	0.20	1.96	1.30	1.07

### 3.2. Patient data

**Figure 6.**
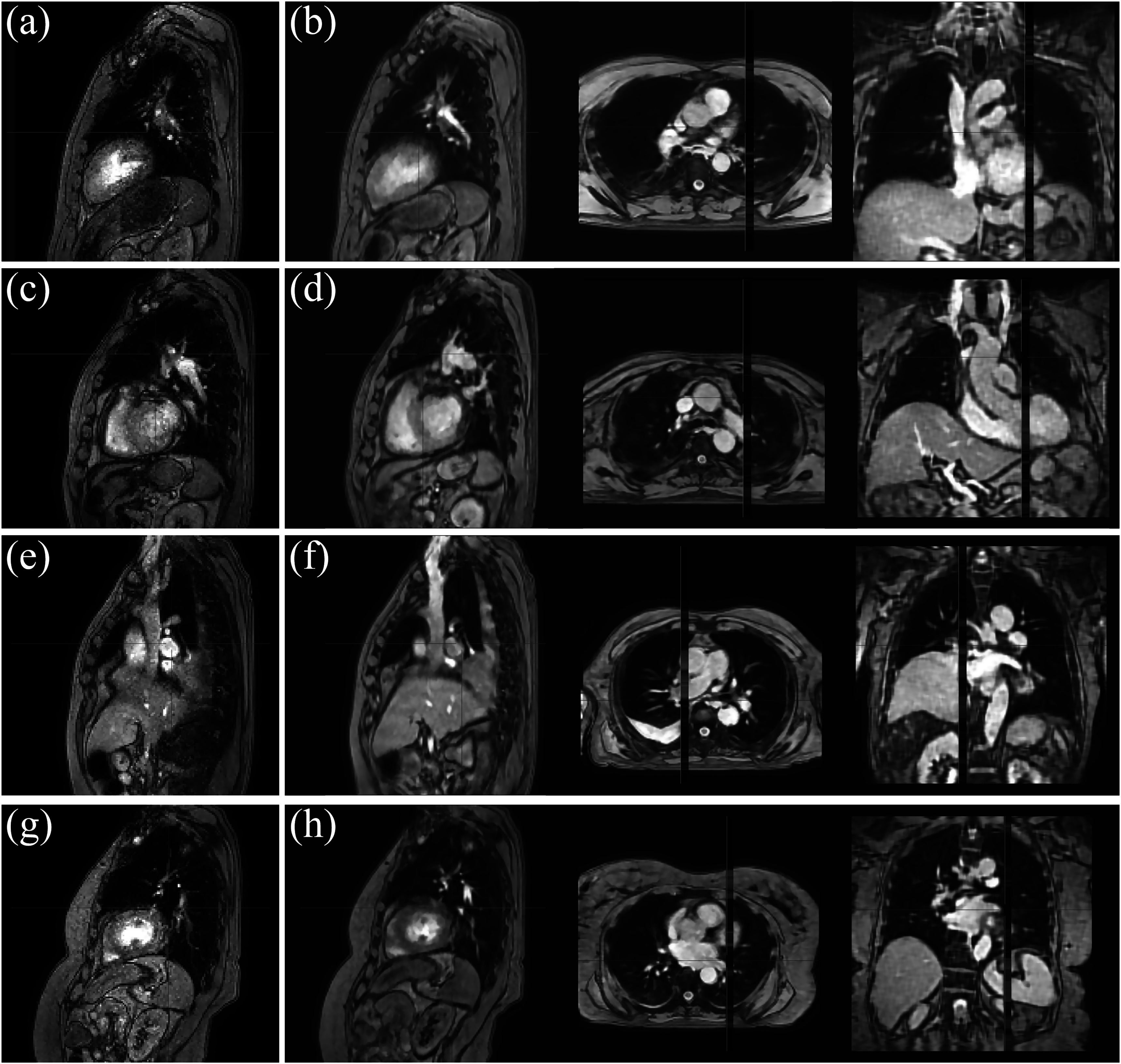
Surrogate images (a), (c), (e), (g) and orthogonal slices of the MCSRIs (b), (d), (f), (h) for patient volunteers one to four. MCSRIs have an isotropic resolution of 2 mm and the 10 mm thick surrogate slice position is shown in red.

Figure 6 shows a surrogate slice and orthogonal slices of the MCSRI for the four patient volunteers. The MCSRIs clearly show many structures with a high spatial resolution in all 3 dimensions and little signs of motion blur, except in the region of the heart. This indicates that the motion models have successfully compensated for the motion and enabled the super resolution reconstruction. Furthermore, the blurring that could be observed at the lower posterior lung-to-rib-cage interface in the simulated data does not appear to be as prominent for the patient datasets.

Table 5 shows the results for the residual error registration. As for the simulated cases, the errors are largest in the SI direction, with a mean value below the voxel resolution of 2 mm except from case p1 (evaluation, day 7) for which the value reaches 2.02 mm.

**Table 5.** Result of the residual error registration for the patient volunteer cases. Results are reported for the building and the evaluation repetitions separately. All results are given in mm.

Residual error registration, patient [mm]											
			mean			std.			95th percentile		
case	phase	day	SI	AP	LR	SI	AP	LR	SI	AP	LR
p1	building	1	0.96	0.79	0.84	0.25	0.23	0.21	1.48	1.29	1.26
p1	evaluation	1	1.83	1.28	1.38	0.45	0.42	0.30	2.67	2.02	1.93
p1	building	6	1.10	0.91	0.97	0.29	0.29	0.31	1.65	1.47	1.43
p1	evaluation	6	1.96	1.47	1.77	0.79	0.51	0.55	3.40	2.42	2.86
p1	building	7	1.14	0.93	0.93	0.34	0.30	0.29	1.80	1.60	1.51
p1	evaluation	7	2.02	1.58	1.54	0.64	0.54	0.49	3.31	2.59	2.60
p2	building	1	1.12	0.96	0.93	0.30	0.31	0.19	1.75	1.73	1.29
p2	evaluation	1	1.52	1.22	1.20	0.32	0.37	0.24	2.10	1.98	1.65
p2	building	5	1.12	0.97	0.89	0.32	0.35	0.19	1.76	1.71	1.17
p2	evaluation	5	1.56	1.41	1.32	0.44	0.53	0.27	2.43	2.51	1.80
p2	building	6	1.16	0.98	0.90	0.38	0.43	0.18	1.99	1.94	1.25
p2	evaluation	6	1.68	1.31	1.36	0.41	0.49	0.29	2.41	2.39	1.85
p3	building		1.12	0.98	0.85	0.38	0.41	0.19	1.92	1.89	1.21
p3	evaluation		1.49	1.33	1.09	0.43	0.62	0.28	2.31	2.64	1.69
p4	building		0.86	0.77	0.73	0.20	0.21	0.15	1.26	1.17	1.02
p4	evaluation		1.09	0.96	0.95	0.21	0.26	0.18	1.48	1.45	1.33

The 95th percentiles are generally larger than for the simulations, but are still 3.40 mm or less. The residual errors are lower for the data used to fit the models than for the unseen data used for evaluation. The mean errors among all patient cases are 1.07 mm, 0.91 mm, and 0.88 mm in SI, AP, and LR direction respectively for the data used to fit the models. These measures increase to 1.64 mm, 1.32 mm and 1.33 mm for the data used to evaluate the models, an increase between 45% and 53%. In summary, the results are better on the building data than on the evaluation data and are slightly worse for the patient data than for the simulated data. Moreover, we can infer from the results on the simulated data and the residual error results on the real data that the models on the real data likely have a mean DFE in each direction of less than 2 mm and a 95th-percentile error of less than 4 mm on the evaluation datasets.

## 4. Discussion

We presented a surrogate-driven motion model, its open-source implementation, and application for MR-guided radiotherapy. To tailor the widely applicable motion modelling framework towards lung cancer treatments on an MR-Linac, we developed an acquisition scheme that utilises a series of 2D images to generate the surrogate signals as well as the motion data. We furthermore evaluated the models’ performance by the means of geometric accuracy on simulated and patient datasets.

An outstanding feature of the motion modelling framework is its ability to fit a model directly to the input data thus avoiding sorting artefacts such as missing or repeated structures. This made it possible to use 2D cine data to sequentially acquire full anatomical and motion information. Furthermore, the reconstruction of the high resolution MCSRI can be performed at the same time and thus the output of our framework contains both, anatomical and motion information.

The motion estimation provided by the model which is also used for compensating for motion during image reconstruction was very effective and the ground-truth anatomy could be recovered with high fidelity. While the geometric accuracy of the models was generally high, they also showed a decrease in the geometric accuracy between building and evaluation data. The mean error measured by residual error registration for the patient data increased between 45% and 53% to 1.62 mm, 1.32 mm and 1.33 mm on average in SI, AP, and LR direction respectively. While it can be expected that any model better fits the data on which it was built, it also means that further investigations will be necessary as to how long the proposed model will be valid for. Baseline drifts may occur during the delivery time of radiotherapy (Takao *et al* 2016). A substantial change in the temporal average position of a patient’s internal organs could impact the accuracy of the proposed models. But none of the patient volunteers in this study showed drifts during the ≈8 min evaluation phase to an extent that final conclusions in this regard can be drawn. However, the required duration of validity will be determined by the integration of the model into a specific workflow. The mean residual error however stayed, with one exception, within the voxel resolution of the MCSRI, i.e. 2 mm.

Sliding motion appeared to be a source of error in the motion estimation provided by the model, with a more obvious impact on the simulated data. This can be attributed to the B-spline basis of our transformation model. Here, sliding can only be approximated by local shear due to continuity assumptions in the transformation formulation. The extended spatial support of the B-spline basis function may be a disadvantage with respect to sliding motion. However, its extended support appears to be advantageous during the model fitting because it effectively integrates information from the separate 2D slices when calculating the gradient during model fitting. Since non-parametric transformation models—such as elastic, fluid, or optical flow—do not share this property, the B-spline transformation has a conceptual advantage. In this regard, implementing a sliding B-spline transformation as proposed by Eiben *et al* (2018) could potentially improve the performance at the sliding interfaces in the future. While the XCAT phantom is a valuable research tool to simulate reasonably realistic respiratory motion, including for instance sliding and hysteresis, it cannot be expected to accurately mimic sudden motion observed in real patients, such as coughing and sneezing. This limits its utility to evaluate velocity-related effects.

Evaluation of the proposed modelling framework on patient datasets was limited to eight datasets from four patients who volunteered to take part in additional scanning sessions on the Elekta Unity MR-Linac. While this enables evaluation of the feasibility or our framework, robustness for a specific clinical implementation will need to be demonstrated on a larger patient cohort in the future.

The acquisition scheme was developed with a flexible implementation into a clinical workflow in mind. It provides the model with the required surrogate and motion data and could be acquired pre-treatment as well as during delivery. The surrogate signals were derived from a slice positioned centrally on the tumour, which enables continuous monitoring of the target motion in the sagittal plane. Hence, in one implementation scenario the acquisition scheme could be used only prior to treatment to build a model. Then during treatment only surrogate slices are acquired to estimate the 4D motion. Since our surrogate slices are equivalent to online cine images currently being used for motion monitoring in clinical MR-Linac treatments, an integration with existing motion mitigation strategies such as gating or tracking is straight forward. Hence the 2D images centred on the tumour could be used twofold: first to guide online-treatment, and second surrogate signals can be calculated from these to apply the fitted model. Various methods to estimate motion of the radiation target in the imaged plane have been implemented and compared previously (Bjerre *et al* 2013, Sawant *et al* 2014, Paganelli *et al* 2015, Zachiu *et al* 2015, Seregni *et al* 2016, Fast *et al* 2017). Whilst additional slices—set to be orthogonal to the previous and also centred on the tumour—may provide information about the centroid target motion, deformations occurring outside the image planes remain undetectable. The volumetric motion data could however be provided by our motion model which could be utilised for retrospective or online 3D dose reconstruction. In another scenario, the full acquisition scheme could also be used during treatment delivery, i.e. acquiring motion slices as well as surrogate slices. This would provide a potentially more accurate estimate of the motion for offline dose reconstruction after treatment has ended to inform, for instance, inter-fraction treatment adaptation.

Any online treatment scenario would furthermore require continuous evaluation of the model’s accuracy. This can be achieved by comparing online acquired data with the corresponding model estimate. A suitable comparison method will depend on the application but could, for instance, be a simple image similarity metric or the results from the residual error registration. While these scenarios highlight some possible implementation strategies, integration into a specific clinical workflow will be part of future work.

Average lung tumour motion amplitudes in the SI direction were measured using implanted fiducials and orthogonal fluoroscopic kilo-voltage imaging for instance by Seppenwoolde *et al* (2002), or Dhont *et al* (2018) to be 12 ± 2 mm and 16.4 ± 7.6 mm respectively. To cover the associated uncertainty of the target position during delivery the geometric concept of the planning target volume (PTV) was introduced. Van Herk *et al* (2000) derived an analytical formulation to calculate margin size around the CTV to achieve adequate target coverage. In this formulation, unmitigated motion is considered a random error, resulting in dose blurring and a large CTV-to-PTV margin. Reducing the target position uncertainty could in principle give room for tighter margins. However, if the 4D motion estimates provided by the motion model has an advantage over methods applied to the 2D cine images in order to guide motion mitigation—each method being associated with their own uncertainties—will be a topic to be addressed in the future. Green *et al* (2018) for instance highlight the importance to characterise the system’s accuracy as a whole, hence, any specific integration strategy will need to be evaluated carefully.

Getting time-resolved 4D MR images with a spatial and temporal resolution sufficient for online treatment guidance and adaptation on an MR-Linac is challenging and an active area of research (Stemkens *et al* 2018). Motion models have been proposed to estimate the full volumetric motion from data that can easily be acquired during treatment delivery, such as 2D slices. Thus, a pre-treatment 3D image can be deformed using the model to estimate the full 3D anatomy at any point during treatment delivery. One method to build motion models is using binned respiratory correlated data from CT or MR imaging. The main modes of motion are extracted from the phase images by performing deformable image registration between the phases and then applying PCA on the resulting DVFs (Harris *et al* 2016, Stemkens *et* *al* 2016, Borman *et al* 2019). Garau *et al* (2019) followed a similar approach but refined it to allow for differential models between two separate ROIs. While PCA based models can potentially model variable breathing motion, a major limitation of these approaches is that they fit the models to respiratory correlated images. As the image sorting methods assume reproducible motion, the images provide no information on the inter-cycle variation, and will often contain sorting artefacts if there is considerable variation during the image acquisition.

Huttinga *et al* (2022) proposed a method called real-time Model-based Reconstruction of MOTion from Undersampled Signal (MR-MOTUS) which also splits the problem of obtaining temporally resolved MR data into an offline preparatory phase and an online phase. Motion fields are decomposed into a spatial and a temporal component where, during the online phase, only the temporal component is reconstructed to address the overall latency requirements. Similar to all aforementioned methods from the literature, the spatial component is computed from respiratory correlated data. In a subsequent publication by Huttinga *et al* (2023) they utilise Gaussian processes to facilitate high-speed inference of 3D displacements but still utilise motion models based on respiratory correlated data in their processing. Thus, our method has a conceptual advantage over such previous approaches. Notably, however, the first work published under the MR-MOTUS framework (Huttinga *et al* 2020) fit motion models directly to k-space data and does not require respiratory correlated volumes, but requires very long computation times.

Model fitting times for our algorithm are still a limitation, too. To fit models and reconstruct the MCSRIs took on average about 40 min. However, our implementation is designed for research purposes and was not optimsied for performance. Previous work by Modat *et al* (2010) has shown speed-up factors of about ten times by moving computations to the graphics processing unit (GPU). Many of the processing steps of our algorithm are closely related to the referred work, and we expect a comparable speed up to be achievable by using GPUs in our framework. Computational times further depend on the amount of data used for building the model, but determining the amount of data required in a specific workflow is beyond the scope of this paper. Furthermore, in a fractionated radiotherapy workflow, a model built and an image reconstructed in a previous fraction may be used in different ways to reduce computational times. For instance, a previously built model could be used to initialise the fitting procedure. If inter-fractional anatomical changes are small, a previously reconstructed MCSRI could be used and adapted to the current fraction by applying a constant deformation offset. This would eliminate the requirement of reconstructing a new image from scratch. Along the same lines, implementation into a specific clinical workflow may benefit from adaptation of individual components presented here. For instance, if only accurate motion estimates would be required and a high-resolution image is not essential, a simpler, non-iterative image reconstruction may be sufficient to fit the model. All these potential adaptations can be expected to decrease the computational time required before the model can be applied. The flexibility of the presented framework will be evaluated in this regard in the future. Application of the motion model is very fast and in the order of 400 ms including surrogate signal generation, DVF generation, and image transformation. To gear the model further towards an online real-time application, predict-ahead methods could be applied to the surrogate signals to account for overall system latency. For this we could build on over a decade of prediction methods developed for respiratory signals (see e.g. Jöhl *et al* (2020) for an extensive comparative study) and is thus beyond the scope of this paper.

## 5. Conclusions

The unified image registration and motion modelling framework was implemented as an open-source software package and successfully tailored towards an MR-Linac workflow for lung-cancer radiotherapy. An acquisition scheme was developed based on thick 2D slices as building blocks, enabling a flexible, staged integration into a future clinical workflow. The model accurately reconstructed the anatomy as well as the respiratory motion from the input data. The geometric accuracy of the models’ motion prediction was measured for simulated cases with known ground truth and for patient cases and the mean error was below the voxel resolution of 2 mm.

## Figures and Tables

**Figure A1.**
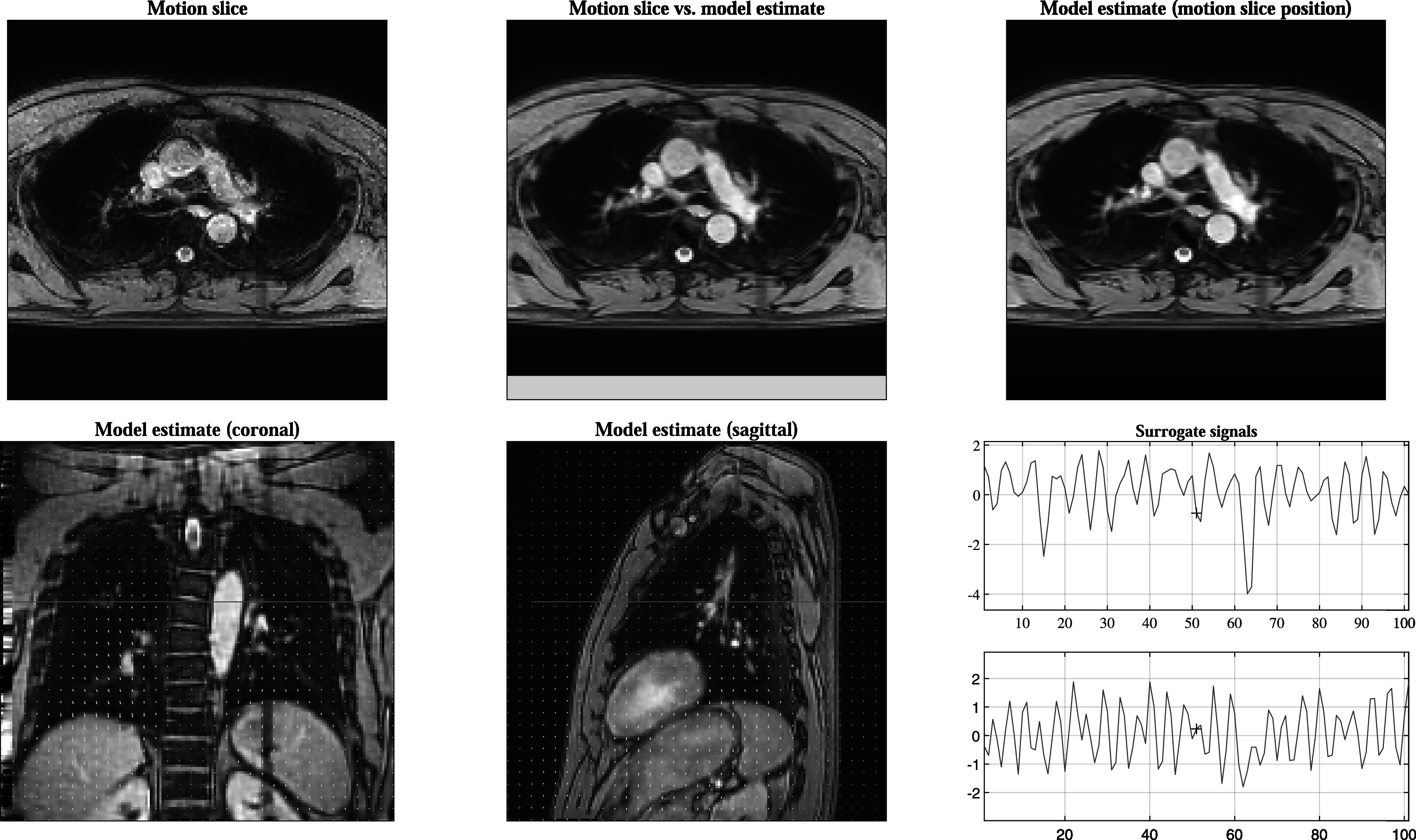
Example frame from the animations available as supplementary material (patient dataset p1, day 1).

## Data Availability

The data cannot be made publicly available upon publication because they contain sensitive personal information. The data that support the findings of this study are available upon reasonable request from the authors.

## Ethical statement

Patients were consented under the PRIMER study, approved under IRAS: 208 449 and REC: 17/LO/0907. This implies that the Declaration of Helsinki and all local statutory requirements and regulations were obeyed.

## Appendix

### A.1. Motion model fitting and image reconstruction parameters

The parameters to perform the model building were selected as follows and details are given below:

-sx 10 -be 0.9 -dType 1 -mcrType 3 -maxMCRIt 1 -maxSwitchIt 10 -maxFitIt 50

The B-spline FFD transformation was set to a control point grid spacing of 10 mm (-sx 10) on the finest of three multi-resolution levels (default value). A bending energy penalty term with a weight of 0.9 was selected (-be 0.9) and thick slices were set as the input data type (-dType 1). From the input data the MCSRI was calculated using iterative back-projection (-mcrType 3) where the reconstruction is continually updated from each reconstruction step, with each reconstruction step performing just one iteration of the super-resolution reconstruction algorithm (-maxMCRIt 1). The algorithm switched between model fitting and image reconstruction ten times (-maxSwitchIt 10) and the model fitting was allowed to run for up to 50 iterations (-maxFitIt 50).

### A.2. Residual error registration paramters

The parameters to perform the residual error registrations were selected as follows:

-vel -sx 5 --lncc 5.0 -be 0.002 -le 0.02

In detail this means that a stationary velocity field was used to achieve a diffeomorphic transformation (-vel) based on a control-point grid with an isotropic spacing of 5.0 mm (-sx 5.0). Local normalised cross correlation was used as a similarity measure with a Gaussian filter kernel size of 5.0 mm (--lncc 5.0). For regularisation, bending energy (-be 0.002) and a first order penalty (-le 0.02) were used with respective relative weights of 0.002 and 0.02.

### A.3. Animated motion model results

Figure A1 shows a frame from the animations generated for the first simulated dataset (tumour position A, file name model_result_simulationA.mp4) and all patient volunteer datasets (file names model_result_p ^*^ .mp4). The first row shows the acquired motion slice on the left and the model estimate (i.e. the deformed MCSRI using the fitted model and the corresponding surrogate signal) on the right for the same geometric position as the motion slice. The middle image is a colour overlay of the left and right image. Cyan and magenta highlight areas where the model estimate differs from the motion slice. A perfect match would result in no colours being visible. The bottom row shows the model estimate for fixed coronal and sagittal slice locations. Overlaid on these images are the deformation vector fields as green arrows and the location of the current motion slices indicated as a red line. The surrogate signals are shown in the bottom right two figures. Note, due to file size limitations of the journal only the first 60 s are included in the uploaded animation files.
